# Establishment of *Kluyveromyces marxianus* as a Microbial Cell Factory for Lignocellulosic Processes: Production of High Value Furan Derivatives

**DOI:** 10.3390/jof7121047

**Published:** 2021-12-07

**Authors:** Marlene Baptista, Joana T. Cunha, Lucília Domingues

**Affiliations:** CEB—Centre of Biological Engineering, University of Minho, Campus de Gualtar, 4710-057 Braga, Portugal; marlenebq16@gmail.com (M.B.); jcunha@ceb.uminho.pt (J.T.C.)

**Keywords:** *Kluyveromyces marxianus*, lignocellulosic biorefinery, furfural, HMF, furfuryl alcohol, 2,5-bis(hydroxymethyl)furan, whole-cell biocatalysis

## Abstract

The establishment of lignocellulosic biorefineries is dependent on microorganisms being able to cope with the stressful conditions resulting from the release of inhibitory compounds during biomass processing. The yeast *Kluyveromyces marxianus* has been explored as an alternative microbial factory due to its thermotolerance and ability to natively metabolize xylose. The lignocellulose-derived inhibitors furfural and 5-hydroxymethylfurfural (HMF) are considered promising building-block platforms that can be converted into a wide variety of high-value derivatives. Here, several *K. marxianus* strains, isolated from cocoa fermentation, were evaluated for xylose consumption and tolerance towards acetic acid, furfural, and HMF. The potential of this yeast to reduce furfural and HMF at high inhibitory loads was disclosed and characterized. Our results associated HMF reduction with NADPH while furfural-reducing activity was higher with NADH. In addition, furans’ inhibitory effect was higher when combined with xylose consumption. The furan derivatives produced by *K. marxianus* in different conditions were identified. Furthermore, one selected isolate was efficiently used as a whole-cell biocatalyst to convert furfural and HMF into their derivatives, furfuryl alcohol and 2,5-bis(hydroxymethyl)furan (BHMF), with high yields and productivities. These results validate *K. marxianus* as a promising microbial platform in lignocellulosic biorefineries.

## 1. Introduction

The yeast *Kluyveromyces marxianus* has been emerging as an alternative cell factory to produce ethanol, high-value chemicals, and enzymes with a wide range of applications in food, feed, and pharmaceutical industries [1]. Due to its Qualified Presumption of Safely (QPS) and Generally Regarded as Safe (GRAS) status, the features of *K. marxianus* have rendered it as an attractive chassis for different industrial applications. These features include: a high growth rate among other eukaryotes [2], thermotolerance (the ability to grow at temperatures up to 52 °C), tolerance to low pH [3], the ability to metabolize a broad range of sugar substrates (glucose, xylose, lactose, fructose, arabinose, galactose, among others), and the ability to produce lytic enzymes [4]. Accordingly, *K. marxianus* application in a biorefinery context is gaining special interest due to its ability to ferment various low-cost feedstocks, such as cheese whey, fruit peels, and the sugars derived from lignocellulosic biomass.

Lignocellulosic-based biorefineries have been developed and are suggested to be environmentally sustainable alternatives to replace the use of fossil fuels to produce biofuels and value-added chemicals. One of the challenges associated with the implementation of these biorefineries relies on the inhibitory compounds present in the hydrolysates after the pretreatment or hydrolysis steps of the biomass. These compounds include acetic acid, furfural, 5-hydroxymethylfurfural (HMF), 4-hydroxybenzaldehyde, syringaldehyde, catechol, and vanillin, that cause the inhibition of cell growth and microbial fermentation [5]. Even though there has been an increased usage of *K. marxianus* in lignocellulose-to-ethanol processes, the knowledge of its stress physiology to multiple inhibitor resistance in lignocellulosic hydrolysates is still poor compared to *Saccharomyces cerevisiae*. Regarding the aldehyde compounds, *K. marxianus*, as *S. cerevisiae*, is known to reduce them into their corresponding alcohols to reduce their toxicity [6], being capable of producing ethanol in the presence of 10 mM of HMF or furfural [7]. Furthermore, *K. marxianus* was identified as an HMF tolerant yeast among several non-conventional yeasts [8]. In fact, a *K. marxianus* strain isolated from a Mezcal fermentation process was found to have a higher tolerance to HMF and furfural than the commercial *S. cerevisiae* Ethanol Red strain [9].

Despite being generally considered as undesirable inhibitors in lignocellulosic processes, HMF and furfural (obtained from dehydration of glucose and xylose, respectively) were identified as the top promising compounds to be obtained from biomass to reach economically viable biorefineries [10]. These furans present a versatile composition—an aromatic furan ring and reactive functional groups (aldehyde group in furfural; aldehyde and alcohol groups in HMF), which makes them promising building-block platforms that can be converted into a wide variety of compounds with applications in diverse areas, such as plastic, pharmaceutical, and textile industries [11,12].

Up until now, the production of these higher-value derivatives has been mainly based on chemical catalysis, with drawbacks such as harsh reaction conditions, expensive catalysts, and low selectivity [13]. More recently, biocatalysis has appeared as a more environmentally friendly alternative, with the use of whole-cell biocatalysts presenting advantages over the use of purified enzymes, e.g., the easiness of catalyst recycling and the regeneration of cofactors [14]. Following that, some microorganisms have been reported to be used in the bioconversion of HMF and furfural into their higher value derivatives, with the production of their corresponding alcohols, 2,5-bis(hydroxymethyl)furan (BHMF) and furfuryl alcohol, receiving great attention [11,12,15]. Furfuryl alcohol has been used as a precursor mainly to produce resins for the foundry industry, but also as an intermediate to produce diuretic furosemide, ranitidine, an antiulcer drug, and chloroquine, used in the treatment of malaria. Moreover, furfuryl alcohol has applications in the wood industry to produce impregnating agents, resins, or adhesives [16]. In turn, BHMF has been studied for its application in the synthesis of polymers, resins, and ethers for the replacement of phthalate plasticizers or the production of polyesters [17,18,19].

Considering this, and the necessity to reach economically viable biorefineries through the production of both bioethanol and high-value compounds [20,21], in this work, we aimed to (1) evaluate the profile of industrial *K. marxianus* isolated from cocoa fermentation processes in terms of xylose consumption and tolerance towards lignocellulosic-derived inhibitors, and (2) explore the more stress-tolerant *K. marxianus* isolate as a whole-cell biocatalyst to produce high-value HMF- and furfural-derivatives (BHMF and furfuryl alcohol).

## 2. Materials and Methods

### 2.1. Strains and Growth Conditions

Eight *K. marxianus* strains isolated from cocoa fermentation [22] (S2, S3, S6, S7, S8, S9, S10, and S11) were used in this work. CBS6556, a laboratory strain (ATCC26548), was used as a reference. The strains were maintained on YPD agar plates containing 10 g/L yeast extract, 20 g/L bactopeptone, 20 g/L agar, and 20 g/L D-glucose. For every growth experiment, the strains were precultured overnight at 37 °C and 200 rpm in an orbital shaker in YPD or YPX (10 g/L yeast extract, 20 g/L bactopeptone, and 20 g/L D-xylose), accordingly with the sugar present in the growth medium. All experiments were performed at 37 °C in 250 mL Erlenmeyer flasks with cotton stoppers at 200 rpm in an orbital shaker, and the inoculum was at an initial OD_600nm_ of 0.1.

The initial screening to evaluate the capacity of the strains to metabolize xylose was performed in YPX medium. To evaluate the tolerance to lignocellulosic-derived inhibitors, the strains selected from the initial screening (S8, S9, and S11) and CBS6556 were grown in YPX with 4.8 mM HMF, 7.3 mM of furfural, and 2.8 g/L of acetic acid at pH 5.0, common concentrations present, as an example, in wheat straw hydrolysates [23] with some modifications. The strains S9 (the most robust) and CBS6556 (reference) were tested with higher concentrations of inhibitory compounds in YPX and YPD mediums with 47.6 mM of HMF, 20.8 mM of furfural, or a combination of 23.8 mM of HMF and 10.4 mM of furfural, similar to those reported by Flores-Cosio et al. (2018), which are the highest reported for testing *K. marxianus* tolerance to lignocellulosic-derived inhibitors [9]. Moreover, to evaluate furan derivatives produced by industrially isolated strain S9 and CBS6556, fermentation experiments were conducted for 72 h in YPD medium with 31 mM furfural and 55 mM HMF. Samples were collected over time for further HPLC and UHPLC analysis.

The specific growth rate was calculated as the slope of the natural logarithm of OD_600nm_ corresponding to the exponential growth timepoints.

### 2.2. Furfural and HMF-Reductive Enzymatic Activities

For the analysis of furfural and HMF reducing activities, the *K. marxianus* strains S9 and CBS6556 were grown for 24 h in YPD medium at 37 °C and 200 rpm in an orbital shaker. The cells were collected, and the crude protein extracts were prepared with Y-PER reagent (Thermo Fisher Scientific, Waltham, MA, USA) The total protein concentration was determined using the Pierce™ Detergent Compatible Bradford Assay Kit (Thermo Fisher Scientific).

Furfural and HMF-reducing activities were assayed in each of the cell extracts (in triplicate) by measuring the decrease of NAD(P)H at 340 nm and 30 °C using a microplate reader spectrophotometer. The reaction mixtures were prepared as described by Nilsson et al. (2005) [24]: 100 mM phosphate buffer pH 7.0, 10 mM furfural or HMF, 100 µM of NADH or NADPH, and 20 µL of crude extract (properly diluted). Furfural and HMF reducing activities are expressed as units per mg of protein (U/mg protein), in which units (U) is defined as μmol of NAD(P)H oxidized per min.

### 2.3. Whole-Cell Bioconversion of Furfural and HMF

*K. marxianus* strain S9 (for inoculation) was grown either in YPD or YPX medium (accordingly with the sugar present in the bioconversion medium) at 37 °C for 24 h at 200 rpm in an orbital shaker. The cell suspension was collected by centrifugation for 5 min (at 4000 g and 4 °C), washed, and resuspended in sterile distilled water. Bioconversion assays were performed with 100 g of fresh yeast/L at 37 °C in an orbital shaker at 150 rpm using 100 mL Erlenmeyer flasks (30 mL working volume) with glycerol-locks to create oxygen-limited conditions. The medium used was either YPD with 55.5 mM HMF, YPD with 33.5 or 66 mM of furfural, or YPX with 33.5 or 66 mM of furfural. Samples were collected over time for further HPLC and UHPLC analysis.

### 2.4. Cell Viability Assay

Methylene blue (Merck, KGaA, Darmstadt, Germany) was dissolved in distilled water to a final concentration of 1 g/L. Yeast suspension (0.5 mL) was mixed by vortexing with 0.5 mL of methylene blue solution and examined microscopically in a Neubauer chamber after 3 min on ice. Unstained cells were assumed to be viable.

### 2.5. Analytical Procedures

Samples taken from fermentation assays were analyzed for the quantification of glucose, glycerol, xylose, acetic acid, and ethanol by HPLC using a Bio-Rad Aminex HPX-87H column, operating at 60 °C, with 0.005 M H_2_SO_4_ at a flow rate of 0.6 mL/min. These compounds were detected in a refractive index detector. Furfural, furoic acid, HMF, and HMFCA (5-hydroxymethyl-2-furan carboxylic acid) were also analyzed by HPLC using the same column in the same conditions, except 0.01 M H_2_SO_4_ was used as the mobile phase. In the case of these compounds, the detection was made in a UV detector at 275 nm for furfural, 257 nm for furoic acid, 282 nm for HMF, and 263 nm for HMFCA.

Furfuryl alcohol was quantified by UHPLC using a Shimadzu Nexera X2 UHPLC chromatograph equipped with Diode Array Detector (Shimadzu, SPD-M20A) upon separation in a reverse-phase ZORBAX Eclipse XDBC18 column (4.6 mm × 250 mm, 5 μm), operating at 25 °C, with 50% (*v*/*v*) acetonitrile/water at a flow rate of 1 mL/min. BHMF was also quantified by UHPLC using the same column and eluted at 25 °C with a mixture of acetonitrile/0.4% (NH_4_)_2_SO_4_ (10:90, *v*/*v*) at pH 3.5 and a flow rate of 0.6 mL/min. The peaks corresponding to furfuryl alcohol and BHMF were detected at a wavelength of 213 and 220 nm, respectively.

Yield (%) was defined as the percentage ratio of furfuryl alcohol or BHMF concentration produced to the maximum theoretical amount that can be achieved from the initial amount of furfural and HMF, respectively. Conversion (%) was defined as the percentage ratio of the converted furfural or HMF to the initial amount of these compounds.

## 3. Results

### 3.1. Screening of Different K. marxianus Strains for Xylose Consumption and Tolerance to Lignocellulosic-Derived Inhibitors

**Figure 1:**
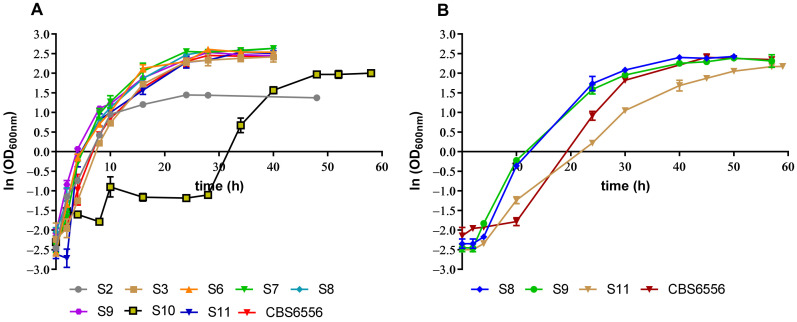
Growth profile of (**A**) different *K. marxianus* strains isolated from cocoa fermentation and laboratory strain CBS6556 in YPX medium and of (**B**) strains S8, S9, S11, and CBS6556 in YPX medium with 4.8 mM of HMF, 7.3 mM of furfural, and 2.8 g/L of acetic acid. Error bars correspond to the standard deviation of two biological replicates.

Considering the *K. marxianus* natural ability to consume xylose, a sugar platform that is present in the liquid fraction of lignocellulosic biomass, we evaluated the potential of different *K. marxianus* strains isolated from cocoa fermentation and the laboratory strain *K. marxianus* CBS6556 to efficiently consume xylose at 37 °C. Growth profiles of the different tested strains are presented in Figure 1A. Since the strains were precultured in YPX medium, the lag phase was practically nonexistent for most strains (Figure 1A). In the case of strain S10, although it displayed the highest specific growth rate during the exponential phase (0.15 h^−1^), this strain presented the longest lag phase (30 h). Strain CBS6556 presented the second highest specific growth rate during the exponential phase (0.13 h^−1^). In turn, strains S8, S9, and S11 presented similar growth rates of approximately 0.10 h^−1^. After 28 h, the growth of most strains entered the stationary phase (Figure 1A), the time at which xylose was fully consumed (data not shown). Nevertheless, strain S10 only totally consumed xylose after 62 h and strain S2 after 38 h. Moreover, strain S2 grew poorly on xylose and never reached optical densities close to the other tested strains. Interestingly, strain S10 was found to be flocculant and only started consuming xylose after 28 h of fermentation. CBS6556 was the strain that produced the lower xylitol concentration (0.40 ± 0.06 g/L) even compared with strains S2 (1.47 ± 0.06 g/L) and S10 (2.88 ± 0.19 g/L), while strains S6, S7, S8, and S9 accumulated the higher xylitol concentration after 37 h of fermentation (4.09 ± 0.05, 4.65 ± 0.02, 4.19 ± 0.30, and 3.84 ± 0.01 g/L, respectively). The ethanol concentration detected for all strains was below 1 g/L.

Since strains S8, S9, and S11 presented similar growth rates in YPX medium, these strains and the laboratory CBS6556 were selected for further evaluation of their tolerance in YPX medium with 4.8 mM of HMF, 7.3 mM of furfural, and 2.8 g/L of acetic acid (pH 5.0). The growth profile of the tested strains is presented in Figure 1B. The lag phase was found to increase 10 h in the presence of the inhibitory compounds, compared to the growth without inhibitors. At the end of fermentation, strain S8 achieved an OD_600nm_ 11.83 ± 0.67 (corresponding to a dry weight of 7.85 g/L), strain S9 achieved 10.15 ± 1.5 (7.70 g/L), strain CBS6556 achieved 9.95 ± 0.30 (7.40 g/L), while strain S11 achieved the lowest OD_600nm_ of 8.78 ± 0.03 (6.70 g/L). Xylose was consumed faster in the case of the strains isolated from cocoa fermentation (50 h), compared to the strain CBS6556 (59 h). Similar to the fermentation in YPX medium alone, strain CBS6556 produced the lower xylitol concentration (0.33 ± 0.05 g/L) after 59 h of fermentation, while the strains S8, S9, and S11 accumulated 6.42 ± 0.08 (after 40 h), 6.29 ± 0.14 (after 40 h), and 7.02 ± 0.10 g/L (after 50 h), respectively. The ethanol concentration detected for the strains isolated from cocoa fermentation was below 0.5 g/L, and it was not detected in the case of strain CBS6556. In the case of strains S8 and S9, both HMF and furfural were detoxified after 10 h of fermentation (data not shown) time at which exponential growth started (Figure 1B), indicating that these strains may present a similar detoxification capacity. On the other hand, strains S11 and CBS6556 only totally detoxified these compounds after 24 h. Approximately half of the acetic acid present in the medium was consumed by strains S8, S9, and S11 after 50 h of fermentation and after 59 h by strain CBS6556 (data not shown).

### 3.2. Evaluation of K. marxianus Strains S9 and CBS6556 Tolerance in the Presence of Higher Inhibitory Loads

**Figure 2:**
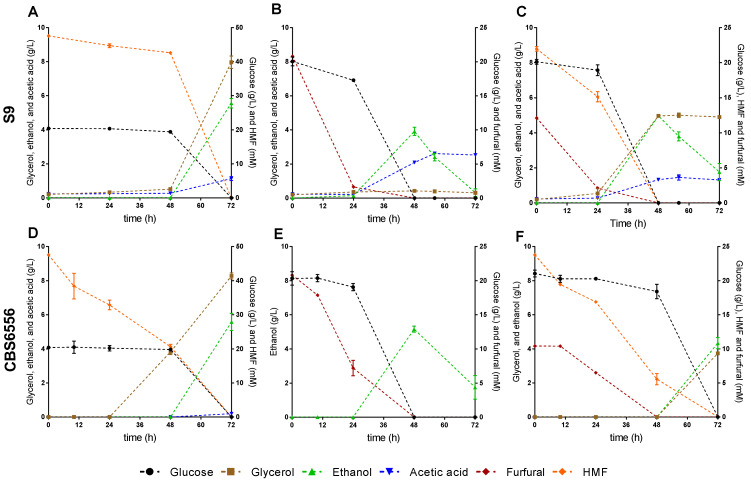
Fermentation profiles of the *K. marxianus* S9 strain isolated from cocoa fermentation and laboratory strain CBS6556 in YPD medium with 47.6 mM of HMF (**A**,**D**), 20.8 mM of furfural (**B**,**E**), or a combination of 23.8 mM of HMF and 10.4 mM of furfural (**C**,**F**). Error bars correspond to the standard deviation of two biological replicates.

Considering that strains S8 and S9 seemed to present a similar detoxification capacity, strain S9 was selected from the previous fermentation with 4.8 mM g/L of HMF, 7.3 mM of furfural, and 2.8 g/L of acetic acid since it proved to be able to cope with these inhibitory compounds and detoxify HMF and furfural after 10 h of fermentation. Furthermore, we decided to test if this strain could cope with a higher concentration of HMF and furfural and compare it with the laboratory strain CBS6556. For this test, both strains S9 and CBS6556 were grown in YPX medium with 47.6 mM of HMF, 20.8 mM of furfural, or a combination of 23.8 mM of HMF and 10.4 mM of furfural. Among the three tested conditions, strain S9 was only able to detoxify 20.8 mM of furfural in 48 h and grow to a final concentration of 4.25 g/L dry weight after 100 h of fermentation (data not shown), while the growth was inhibited in the case of the other conditions. In the case of strain CBS6556, it could not cope with the inhibitory compounds at the concentrations tested, and the growth was inhibited in the three conditions. Furthermore, we evaluated the effect of the same inhibitory loads in YPD medium for the growth and tolerance of both strains S9 and CBS6556. The fermentation profiles for both strains under the tested conditions are presented in Figure 2.

Strain S9 started to detoxify 47.6 mM of HMF after 48 h of fermentation (Figure 2A), detoxified almost 20.8 mM of furfural after 24 h (Figure 2B), and a combination of 23.8 mM of HMF and 10.4 mM of furfural after 48 h (Figure 2C). Glucose was consumed faster in the fermentations with 20.8 mM of furfural and a combination of 23.8 mM of HMF and 10.4 mM of furfural (48 h; Figure 2B, C) than in the fermentation with 47.6 mM of HMF (72 h; Figure 2A). The highest glycerol concentration produced by strain S9 was achieved under the condition with 47.6 mM of HMF (7.97 ± 0.26 g/L; Figure 2A), while lower glycerol concentration was produced by strain S9 in the fermentation with 20.8 mM of furfural (0.31 ± 0.01 g/L; Figure 2B). Strain S9 produced the highest acetic acid concentration during fermentation with 20.8 mM of furfural (2.53 ± 0.07 g/L; Figure 2B), which was more than double of the concentration produced under the other two tested conditions. In the conditions where the lag phase was shorter (20.8 mM of furfural and a combination of 23.8 mM of HMF and 10.4 mM of furfural; data not shown), glucose was converted to ethanol, which, in turn, was used as a carbon source when glucose was totally consumed. On the other hand, in the condition with 47.6 mM of HMF, where the lag phase was longer, ethanol consumption was not observed.

Strain CBS6556 detoxified 47.6 mM of HMF in 72 h of fermentation (Figure 2D), 20.8 mM of furfural in 48 h (Figure 2E), and in the condition with a combination of 23.8 mM of HMF and 10.4 mM of furfural, HMF was detoxified at 72 h while furfural was detoxified in less time (48 h; Figure 2F). Glucose was consumed faster (48 h) in the fermentation with 20.8 mM of furfural (Figure 2E) than in the fermentations with 47.6 mM of HMF and a combination of 23.8 mM of HMF and 10.4 mM of furfural (72 h; Figure 2D,F). The highest glycerol concentration produced by strain CBS6556 was achieved in the fermentation with 47.6 mM of HMF (8.59 ± 0.05 g/L; Figure 2D). Interestingly, glycerol was not detected in the case of the condition with 20.8 mM of furfural (Figure 2E). Acetic acid was only detected under the condition with 47.6 mM of HMF in very low concentration (0.21 ± 0.00 g/L; Figure 2D). In the condition where the lag phase was shorter (20.8 mM of furfural; data not shown), glucose was converted to ethanol, which, in turn, was used as a carbon source when glucose was totally consumed, as observed for strain S9. On the other hand, in the condition with 47.6 mM of HMF or a combination of 23.8 mM of HMF and 10.4 mM of furfural, where the lag phase was longer, ethanol consumption was not observed.

Strain S9 converted 47.6 mM of HMF and 20.8 mM of furfural in less time than CBS6556. Moreover, in the condition with a combination of 23.8 mM of HMF and 10.4 mM of furfural, strain S9 was more efficient at detoxifying these compounds than CBS6556 since it was able to convert both inhibitors simultaneously. In contrast, although CBS6556 was able to detoxify furfural at the same time as strain S9, it was slower in HMF detoxification. Furthermore, furfural detoxification was found to be faster than HMF in both strains, except in the condition with a combination of 23.8 mM of HMF and 10.4 mM of furfural, where strain S9 was able to detoxify both inhibitors simultaneously. Strain S9 produced acetic acid under the three conditions tested, and the highest concentration was detected in the presence of 20.8 mM of furfural, while CBS6556 only produced a low concentration in the presence of 47.6 mM HMF. Glycerol production by each strain was higher in the condition with 47.6 mM of HMF than in the presence of 20.8 mM of furfural. In fact, in the presence of 20.8 mM of furfural, a low concentration of glycerol was detected in the case of strain S9, and it was not detected in CBS6556 fermentation under this condition.

### 3.3. HMF and Furfural Reducing Activity for K. marxianus Strains S9 and CBS6556

**Figure 3:**
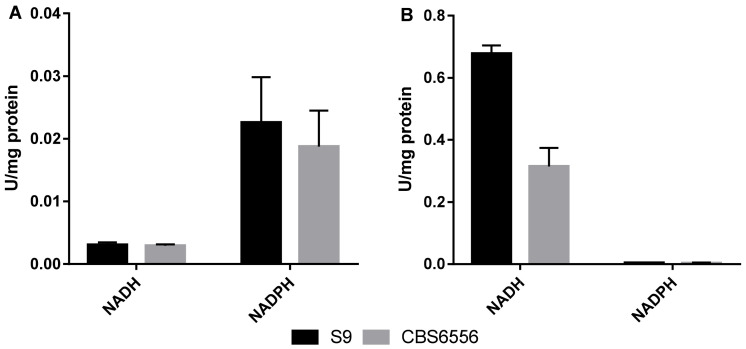
HMF (**A**) and furfural (**B**) reducing activity in crude cell extract for *K. marxianus* S9 strain and laboratory strain CBS6556. Error bars correspond to the standard deviation of three replicates.

We further evaluated the cofactor preference (NAD(P)H) for HMF and furfural reducing activity in strains S9 and CBS6556 (Figure 3).

The average HMF-reducing activity with NADPH as the cofactor was higher than with NADH for both strains. Moreover, HMF-reducing activity with NADPH was slightly higher for strain S9 than for CBS6556 (Figure 3A). On the other hand, the average furfural-reducing activity for both strains was higher with NADH as the cofactor than with NADPH. In addition, furfural-reducing activity with NADH for strain S9 was two-fold higher than that for CBS6556 (Figure 3B).

#### Assessment of Furfural and HMF Derivatives Produced by K. marxianus Strains S9 and CBS6556

**Figure 4:**
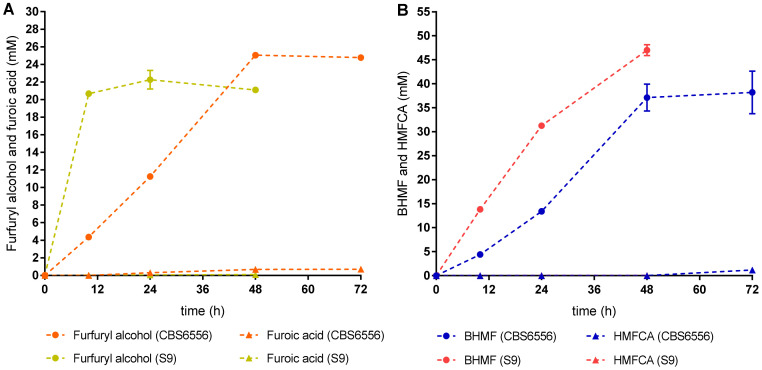
Profile of reduction of 31 mM furfural (**A**) and 55 mM HMF (**B**) into their respective alcohols (furfuryl alcohol and 2,5-bis(hydroxymethyl)furan - BHMF) and carboxylic acids (furoic acid and 5-hydroxymethyl-2-furan carboxylic acid - HMFCA) by *K. marxianus* strain S9 and laboratory strain CBS6556. Fermentation conditions were: YPD medium with 31 mM furfural (**A**) and 55 mM HMF (**B**), 37 °C, 200 rpm, 72 h. Error bars correspond to the standard deviation of two biological replicates.

Considering that both *K. marxianus* strains S9 and CBS6556 were able to grow in YPD medium and cope with a high concentration of HMF and furfural, we decided to assess the furan derivatives resulting from the detoxification of these inhibitory compounds by both *K. marxianus* strains (Figure 4). The conversion of furfural and HMF into their corresponding alcohol, or carboxylic acid in the presence of oxygen, is essential for cell growth and ethanol production since these compounds are less toxic compared to furfural and HMF.

Furfural and HMF were found to be mainly converted by *K. marxianus* strains S9 and CBS6556 to their corresponding alcohols (furfuryl alcohol and BHMF, respectively; Figure 4). *K. marxianus* strain S9 was able to convert furfural into 21.09 ± 0.12 mM of furfuryl alcohol, while strain CBS6556 was able to achieve 24.78 ± 0.25 mM of furfuryl alcohol (Figure 4A). Although the concentration of furfuryl alcohol achieved by strain S9 was lower than that obtained by CBS6556, strain S9 achieved almost the maximum of furfuryl alcohol more readily (at 10 h) than strain CBS6556 (at 48 h), which represents a remarkable difference between the strain isolated from cocoa fermentation and the laboratory strain (Figure 4A). Furfuryl alcohol conversion to furoic acid was very limited in both strains, although strain CBS6556 was able to produce seven times more of this carboxylic acid than strain S9 (0.70 mM by CBS6556 and 0.10 mM by S9; Figure 4A).

*K. marxianus* strain S9 was able to convert HMF into a higher concentration of BHMF (47.01 ± 0.81 mM) than strain CBS6556 (38.21 ± 3.15 mM; Figure 4B). Although the maximum BHMF concentration was achieved by both strains after 48 h, strain S9 reached a higher concentration. As observed in the case of furoic acid, both strains produced low HMFCA concentrations, although strain CBS6556 produced 29 times higher HMFCA concentration (1.19 ± 0.01 mM) than strain S9 (0.04 ± 0.00 mM; Figure 4B).

### 3.4. Whole-Cell Bioconversion of Furfural and HMF by K. marxianus Strain S9

**Figure 5:**
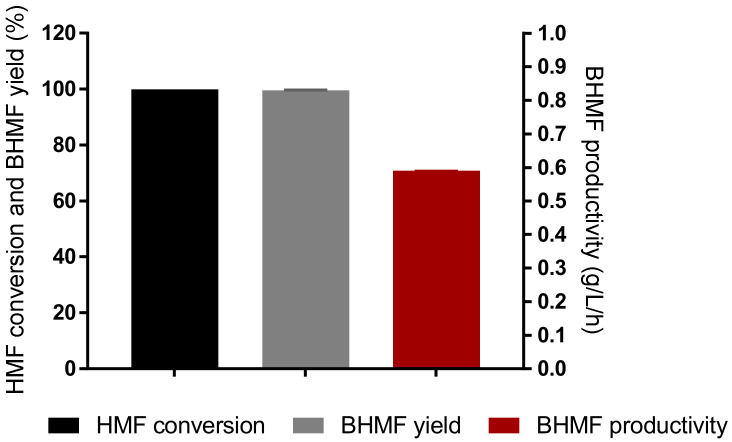
Biocatalytic synthesis of BHMF by *K. marxianus* S9 strain. Reaction conditions: 55.5 mM HMF, YPD medium (111 mM glucose), 100 g/L (wet weight) yeast cells, 37 °C, 150 rpm, 72 h. Values were calculated at the maximum, achieved after 12 h of fermentation. Error bars correspond to the standard deviation of two biological replicates.

**Figure 6:**
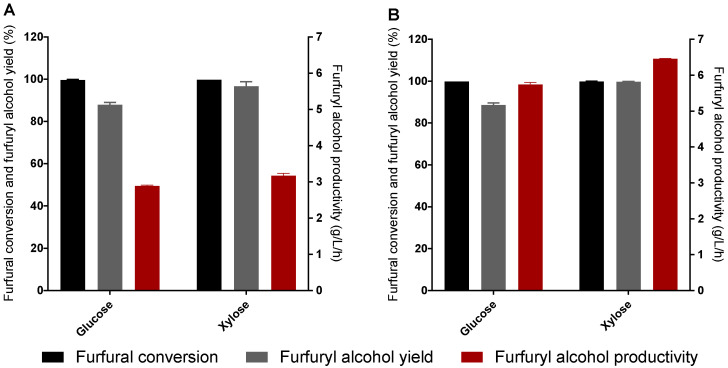
Biocatalytic synthesis of furfuryl alcohol by *K. marxianus* S9 strain. Reactions conditions: (**A**) 33.5 mM furfural, YPD (111 mM glucose) or YPX medium (133 mM xylose), 100 g/L (wet weight) yeast cells, 37 °C, 150 rpm, 72 h; (**B**) 66 mM furfural, YPD (111 mM glucose) or YPX medium (133 mM xylose), 100 g/L (wet weight) yeast cells, 37 °C, 150 rpm, 72 h. Values were calculated at the maximum, achieved after 1 h of fermentation. Error bars correspond to the standard deviation of two biological replicates.

Considering that strain S9 was found to efficiently detoxify both furfural and HMF and convert those compounds into their corresponding alcohol (furfuryl alcohol and BHMF), we further performed bioconversion assays in the presence of 55.5 mM of HMF and glucose (Figure 5) and 33.5 or 66 mM of furfural in the presence of glucose or xylose (Figure 6), aiming to establish strain S9 as a whole-cell biocatalyst for furfuryl alcohol and BHMF production. Furfural conversion to furfuryl alcohol was also tested in xylose medium as furfural is obtained from the dehydration of this pentose, and they are commonly present together in the hemicellulosic hydrolysates of lignocellulosic biomass [14].

Strain S9 was able to achieve maximum conversion of 99.93% of HMF, with a yield of 99.65% and a productivity of 0.59 g/L/h of BHMF after 12 h of fermentation (Figure 5). HMFCA steadily increased, reaching approximately 0.22 mM after 72 h. Glucose was totally consumed after 1 h of the assay (data not shown). After 72 h of fermentation, 94.80% of cells were still viable, indicating that the conditions were not toxic to the cells.

In the presence of 33.5 mM of furfural and glucose as co-substrate, strain S9 was able to achieve a conversion of 99.68% of furfural, with a yield of 87.90% and a productivity of 2.89 g/L/h of furfuryl alcohol after 1 h of fermentation. In the presence of the same furfural concentration but using xylose as co-substrate, strain S9 achieved a slightly higher conversion of furfural (99.81%), with a higher yield (96.68%) and productivity of furfuryl alcohol (3.18 g/L/h) than in the presence of glucose as co-substrate (Figure 6A).

In the presence of 66 mM of furfural and glucose as a co-substrate, strain S9 was able to achieve a conversion of 99.86% of furfural, with a yield of 88.71% and a productivity of 5.74 g/L/h of furfuryl alcohol after 1 h of fermentation. In the presence of the same furfural concentration but using xylose as co-substrate, strain S9 achieved the same conversion of furfural, with a higher yield (99.75%) and productivity of furfuryl alcohol (6.46 g/L/h) than in the presence of glucose as a co-substrate (Figure 6B).

Glucose was totally consumed after 1 h of the assay either in the presence of 33.5 or 66 mM of furfural, while xylose was only consumed after 72 h either in the presence of 33.5 or 66 mM of furfural (data not shown).

Furoic acid concentration steadily increased, reaching approximately 0.25 mM after 72 h in the condition initially with 33.5 mM of furfural, either in the presence of xylose or glucose. In the condition initially with 66 mM of furfural, either in the presence of xylose or glucose, the concentration of furoic acid steadily increased, reaching 0.40 mM after 72 h.

After 72 h of the assay, approximately 91% of cells were still viable in the condition with initially 33.5 mM of furfural and in the presence of glucose, while in the presence of xylose, only 72.8% of the cells were still viable by the end of the assay. In the condition where 66 mM of furfural was used and in the presence of glucose, the percentage of viable cells was also higher (90.9%) than in the presence of xylose (81.8%).

## 4. Discussion

During the pretreatment and hydrolysis steps of the lignocellulosic biomass, several inhibitors are released, such as furfural, HMF, and acetic acid, which can be toxic and hinder microbial fermentation [5]. As such, the search for robust microorganisms able to cope with these inhibitory compounds is of utmost importance to achieve efficient fermentations. The non-conventional yeast *K. marxianus* has emerged as a promising biofactory, currently being mainly explored for ethanol production [25]. Xylose assimilation in *K. marxianus* is accomplished by the xylose reductase/xylitol dehydrogenase (XR/XDH) pathway, where xylitol is the first intermediate, and its accumulation is caused by the cofactor imbalance between XR and XDH enzymes [26,27].

In this work, we explored the growth profile of eight *K. marxianus* strains isolated from cocoa fermentation and the laboratory strain CBS6556 at 37 °C in a xylose-rich medium. Since strains S8, S9, and S11 presented similar growth rates in YPX medium, their stress tolerance to lignocellulosic-derived inhibitors was assessed in the same medium with 4.8 mM of HMF, 7.3 mM of furfural, and 2.8 g/L of acetic acid at pH 5.0. It is worth noting that the pH of the medium in this experiment was adjusted to 5.0 since it is reported that at pH 4.0 or 4.5 *K. marxianus* growth is retarded when acetate is added to the medium [28]. During the lag phase, furfural and HMF were being converted to less toxic derivatives, allowing the exponential growth to start after that period. Strains isolated from cocoa fermentations showed higher tolerance to the inhibitors tested than the laboratory strain. Moreover, the low concentration of ethanol achieved could be a result of the strains only being able to consume half of the acetic acid introduced in the medium, as previously observed by Nitiyon et al. (2016) [7]. Gathering these results, it is evident that *K. marxianus* strain variability is reflected in their ethanol and xylitol production and their stress tolerance, as previously reported by Nitiyon et al. (2016) [7] for *K. marxianus* BUNL-21 and DMKU3- 1042, and by Wilkins et al. (2008) for *K. marxianus* strains IMB2, IMB4, and IMB5 [29], as examples. Recently, Wang et al. (2018) performed a transcriptomic analysis by RNA-seq after *K. marxianus* growth at 42 °C in the presence or absence of a mixture of lignocellulosic inhibitors similar to the ones used in our study (3 g/L acetic acid, 0.7 g/L furfural, 0.7 g/L HMF, and 0.28 g/L phenols). The authors showed that most of the differentially expressed genes from glycolysis, gluconeogenesis, pyruvate metabolism, and NADPH metabolism, among other pathways, were down-regulated, while genes involved in the TCA and pathways involved in stress response were up-regulated (such as alcohol dehydrogenases encoding isoform genes, *ADH3*, *ADH4*, and *ADH6*). This suggests that *K. marxianus* boosted up the mechanisms for the detoxification of these inhibitors [30].

Considering that strains S8 and S9 seemed to present a similar detoxification capacity, strain S9’s ability to cope with a higher concentration of HMF and furfural was tested in YPX medium and compared with the laboratory strain CBS6556. As a result, strain CBS6556 did not grow under any condition, while strain S9 only grew in the condition with furfural alone. Moreover, in the xylose-rich medium, the presence of 10.4 mM of furfural is known to increase acetic acid yield from 0.09 g/g xylose to 0.13 g/g xylose compared to a control without furfural [31]. In our work, strain S9 was able to achieve a higher yield of approximately 0.47 g/g xylose. One of the possible explanations for acetic acid accumulation in YPX is the need for NADPH as a cofactor for xylose reductase, which can be supported by the up-regulation of the *ALD4* gene coding for acetaldehyde dehydrogenase on YPX [7].

We hypothesized that the growth inhibition in YPX was caused by NADPH cofactor competition between xylose reductase, the first enzyme of the oxidoreductive pathway in *K. marxianus*, and the oxidoreductases involved in HMF detoxification [5,27]. To evaluate the effect of carbon sources on the detoxification of HMF, we tested the same inhibitory conditions in YPD medium. As a result, the growth of both strains was not inhibited, and strain S9 proved to be able to detoxify HMF and furfural more readily than the laboratory strain CBS6556. Temperature and ethanol tolerance are attributes that cocoa fermentation strains may display, given that the temperature can reach 40 °C and ethanol can be present at 10–12% under the operation conditions [32]. The strain S9 proved to be able to tolerate 37 °C and was capable of detoxifying a high concentration of inhibitory compounds (furfural and HMF) more readily than CBS6556, revealing its higher potential for application in fermentations with real hydrolysates. *S. cerevisiae* strains isolated from industrial distilleries also showed superior ethanol production than laboratory strains [33] and the importance of selecting the best chassis for the target application has been clearly demonstrated [23].

Under glucose growth, acetic acid is produced from acetaldehyde by aldehyde dehydrogenases to regenerate NADH in the cytoplasm [9]. As furfural detoxification requires NADH, this explains the higher production of acetic acid in the presence of furfural. Glycerol production by both S9 and CBS6556 strains was higher in the condition with 47.6 mM of HMF than in the presence of 20.8 mM of furfural. In fact, in the presence of 20.8 mM of furfural, a low concentration of glycerol was detected in the case of strain S9, and it was not detected in CBS6556 fermentation under this condition. Reports in the literature suggest that furfural detoxification substitutes the formation of glycerol to reoxidize the excess NADH during fermentation and to maintain the intracellular redox balance [34].

Furthermore, an HMF and furfural reducing activity assay corroborated the hypothesis of NADPH cofactor imbalance in YPX medium since NADPH was confirmed to be the preferred cofactor for HMF reduction and NADH for furfural reduction in both strains. Moreover, this assay reinforced that strain S9, isolated from an industrial environment, is more robust than the laboratory strain CBS6556 given its higher furfural and HMF-reducing activity. In *K. marxianus*, NADH regeneration occurs in the second step of the XR/XDH pathway [26], and NADH was proved in our study to be the preferred cofactor for furfural reduction, which explains why only furfural detoxification was possible in the xylose-rich medium. In *S. cerevisiae*, glucose assimilation via the pentose phosphate pathway (PPP) and through the action of glucose-6-phosphate dehydrogenase regenerates NADPH [35]. The same was demonstrated in *Kluyveromyces lactis*, a close yeast to *K. marxianus* [36,37]. As such, assuming that *K. marxianus* uses the same mechanism, this explains why strains S9 and CBS6556 were able to grow in YPD medium and detoxify HMF. Four alcohol dehydrogenases (KmAdh) were found in the *K. marxianus* genome [38], and later its characterization revealed that these enzymes prefer NAD^+^/NADH as cofactors over NADP^+^/NADPH. Specifically, KmAdh1 and KmAdh2 could efficiently reduce furfural using NADH as a cofactor, which corroborates our observations in the furfural reducing activity assay [39]. In another work, a broad specific NADPH-dependent aldehyde reductase, KmGRE2, with a 46% similar identity to the *S. cerevisiae* S288c GRE2 was identified in the *K. marxianus* strain DMB1 and showed 60% more relative activity towards furfural than HMF. The results presented in that study reveal that KmGRE2 could be involved in furfural detoxification [40].

We verified that furfural and HMF were mainly converted by *K. marxianus* strains S9 and CBS65556 to their corresponding alcohols (furfuryl alcohol and BHMF, respectively), and strain S9 was faster than CBS6556 in the production of both alcohols. The low concentrations detected of the corresponding carboxylic acids, HMFCA and furoic acid, were expected since it is reported that under anaerobic conditions, HMF and furfural are mainly converted to their corresponding alcohol while under aerobic conditions, furfural is known to be converted to furoic acid [6,41]. Oliva et al. (2004) evaluated the inhibitory effect of furfural on the growth and fermentation of *K. marxianus* CECT 10875 and further detected the production of furfuryl alcohol at 42 °C in the presence of 30 g/L glucose. Using an inoculum size of 4% (*v*/*v*), the authors showed that 2 g/L (21 mM) of furfural, a concentration that inhibited 25% of the strain growth at 24 h, was completely reduced to furfuryl alcohol after 8 h [6]. In our work, *K. marxianus* strain S9 was able to produce 21.09 mM of furfuryl alcohol in the presence of 20 g/L of glucose after 12 h using an inoculum at OD_600 nm_ 0.1, which is lower than the inoculum used by Oliva et al. (2004).

Finally, considering the demonstrated potential of *K. marxianus* strain S9 to produce furan alcohol derivatives, we performed a whole-cell bioconversion assay under anaerobic conditions and with a higher inoculum to favor the production of the high-value compounds furfuryl alcohol and BHMF. Our results indicate that future experiments for the biocatalytic synthesis of furfuryl alcohol from the xylose-rich liquid fraction of lignocellulosic hydrolysates could be efficient due to the higher conversion, yield, and productivity observed in the presence of xylose (vs. glucose). It also shows the major relevance of the redox balance between xylose consumption and furfural reduction for furfuryl alcohol production. Despite slow xylose consumption during the bioconversion assay, the pre-inoculum performed in YPX probably created an NADH pool necessary for furfural conversion into furfuryl alcohol.

When pulsing furfural during xylose consumption, the redox state and energy metabolism of *S. cerevisiae* cells was found to be more severely affected than during glucose consumption. Therefore, it can be assumed that in *K. marxianus,* furfural addition could cause similar effects, which could explain why cell viability was lower in the assays with xylose than with glucose [42].

There are some reports in the literature describing the production of BHMF by whole-cell biocatalysts. To the extent of our knowledge, the BHMF yield reported in our work (99.65%) is the highest reported for yeast [43,44,45], bacteria [46,47], and fungi [48,49] in batch and fed-batch modes using a synthetic medium with glucose as co-substrate. Specifically, in yeast, Li et al. (2016) reported BHMF production at 35 °C by *Meyerozyma guilliermondii* SC1103 from 100 mM of HMF with an inoculum of 20 g/L of wet weight using glucose as a co-substrate and obtained a yield of 86% [43]. In another work, an *S. cerevisiae* strain harboring an aryl alcohol dehydrogenase from *M. guilliermondii* was reported to produce BHMF with a yield of 94% from 250 mM of HMF with an inoculum of 60 g/L of wet weight using glucose as co-substrate [44].

Furfuryl alcohol production from furfural has also been previously described. To the extent of our knowledge, the furfuryl alcohol productivities presented in our work using glucose as a co-substrate (2.89 g/L/h from 33.5 mM of furfural, and 5.74 g/L/h from 66 mM of furfural) were the highest among the reported for yeast [50,51,52] and bacteria [53,54,55,56,57] in a batch using a synthetic medium. Moreover, the productivities obtained in our work using xylose as co-substrate (3.18 g/L/h from 33.5 mM of furfural, and 6.46 g/L/h from 66 mM of furfural) were also the highest among the reported for yeast [58,59] in a batch using a synthetic medium. For example, Mandalika et al. (2014) reported the production of furfuryl alcohol at 30 °C by *S. cerevisiae* UWOP587-2421 from 25 g/L of furfural with an inoculum of 10 g/L of wet weight using glucose as a co-substrate and achieved a productivity of 0.96 g/L/h [50]. Recently, Kılmanoğlu et al. (2021) intended to optimize the pretreatment and enzymatic hydrolysis conditions of tomato pomace, ultimately aiming to produce alcohols and esters by *K. marxianus* at 28 °C and using a 5% seed culture. The authors were able to produce 0.28 g/L of furfuryl alcohol with a productivity of 0.01 g/L/h. Although low productivity and a final concentration of furfuryl alcohol was obtained in the work by Kılmanoğlu et al. (2021) and also considering that the main goal of the work was not the production of furfuryl alcohol, the authors presented evidence that *K. marxianus* can produce furfuryl alcohol from furfural from an inexpensive real hydrolysate [60].

## 5. Conclusions

In conclusion, our results evidence the higher potential for the application of industrial isolates in lignocellulosic biorefineries compared to laboratory strains, as a result of their higher tolerance to lignocellulosic-derived inhibitors. To the extent of our knowledge, this is the first study exploiting *K. marxianus* as a whole-cell biocatalyst to produce furfuryl alcohol and BHMF. Furthermore, as far as we know, the furfuryl alcohol productivities presented in our work using glucose (5.74 g/L/h) or xylose as a co-substrate (6.46 g/L/h) are the highest reported for yeast, and the 99.65% BHMF yield attained is the highest reported in the literature. Moreover, given the high cell viability at the end of the bioconversion assays, further experiments with cell recycling in a fed-batch scheme and using HMF or furfural-enriched medium obtained from renewable carbohydrates (e.g., lignocellulosic biomass) should be performed to establish a sustainable process to produce furfuryl alcohol and BHMF by *K. marxianus*. On the other hand, the production of these high-value compounds will greatly impact the economic feasibility of lignocellulosic biorefineries.

## Data Availability

Not applicable.
